# Optical, Tomographic, and Mass Spectrometry Imaging Methods for Burn Wounds: Capabilities, Limitations, and Clinical Potential

**DOI:** 10.3390/biomedicines14061223

**Published:** 2026-05-28

**Authors:** Dmitry P. Krylov, Dariya M. Badanina, Dmitry S. Kozlov, Peter S. Timashev, Daria S. Kuznetsova, Artem M. Mozherov

**Affiliations:** 1 Institute for Regenerative Medicine, Sechenov First Moscow State Medical University (Sechenov University), 8-2 Trubetskaya Str., 119991 Moscow, Russia; 2 Department of Biological Chemistry, Sechenov First Moscow State Medical University (Sechenov University), 8-2 Trubetskaya Str., 119991 Moscow, Russia

**Keywords:** thermal injury, multimodal imaging, histopathological analysis, ultrasonography, immunohistochemical analysis, elastography, fluorescence microscopy, fluorescence lifetime imaging microscopy, indocyanine green angiography, magnetic resonance imaging, mass spectrometry-based imaging

## Abstract

This review systematizes the principal methods for imaging and morphological analysis of burn wounds, ranging from light, electron, and fluorescence microscopy to tomographic techniques and mass spectrometry imaging. Light microscopy with histological staining and immunohistochemistry remains the morphological gold standard, enabling visualization of the zones of coagulation, stasis, and hyperemia, as well as molecular characterization of inflammation, angiogenesis, and fibrosis. Electron microscopy allows the study of the ultrastructure of cells and the extracellular matrix at nanometer resolution. Among optical methods, wide-field indocyanine green angiography demonstrates high accuracy in burn depth stratification, whereas fluorescence lifetime imaging microscopy assesses cellular metabolism without exogenous labels. Among tomographic techniques, high-frequency ultrasound is the most accessible bedside modality with submillimeter resolution, permitting evaluation of tissue anatomy, perfusion, and biomechanical properties. magnetic resonance imaging is limited by its high cost and long examination time, while mass spectrometry imaging is used solely for research purposes. For clinical practice, the optimal combination is high-frequency ultrasound and wide-field fluorescence imaging. All methods retain high relevance for experimental research, enabling the validation of novel therapeutic strategies.

## 1. Introduction

Skin is the largest organ of the human body and plays a crucial role in numerous vital functions. It acts as a barrier, regulating body temperature, providing sensory information, supporting the immune system, and helping maintain fluid–electrolyte balance [1]. Skin consists of three structural and functional layers: the epidermis, which provides protection and self-renewal; the dermis, responsible for nutrition, innervation, and thermoregulation; and the hypodermis, which performs thermal insulation and mechanical cushioning. Despite its high regenerative potential, skin remains vulnerable to aggressive environmental factors. Particularly, severe injuries occur from thermal injury, accompanied by disruption of barrier function, vascular regulation, and immune response [2]. Burn injuries represent one of the most clinically significant forms of skin damage and are associated with high mortality, disability, and substantial economic costs. According to the World Health Organization, approximately 11 million burn injuries and about 180,000 deaths are recorded annually [3]. Data from the Global Burden of Disease Study (2021) underscore the substantial global burden of burns, with an estimated 12.99 million severe cases and 235.34 million mild cases worldwide. Severe burn injuries may progress to burn shock and burn disease, characterized by systemic inflammatory response, metabolic disorders, and multiple organ dysfunction. Thus, even with high survival rates, patients often face prolonged rehabilitation, scarring, chronic pain, infectious complications, and long-term impairment of quality of life [4].

**Figure 1:**
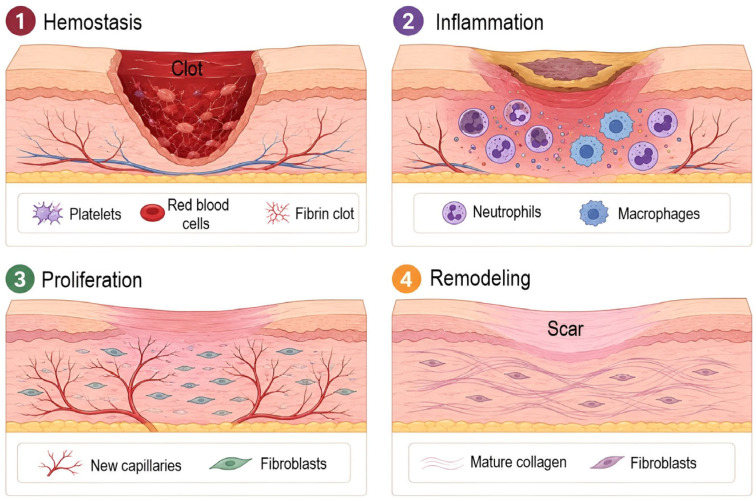
Stages of burn wound healing. Generated with ChatGPT (OpenAI, GPT-5 with image generation capabilities) and manually edited by the authors.

Under physiological conditions, skin possesses a high regenerative capacity, and the healing of acute wounds typically takes approximately 8–12 weeks. This process is a coordinated mechanism involving interactions among cellular populations (keratinocytes, fibroblasts, and endothelial cells), signaling molecules (growth factors, cytokines, and chemokines), and extracellular matrix components. Traditionally, wound healing proceeds through four phases that partially overlap in time: hemostasis, inflammation, proliferation, and remodeling [5]. The essence of the hemostasis phase is the formation of a platelet–fibrin clot, which also initiates the subsequent stages of regeneration. The inflammatory phase is characterized by the migration of neutrophils and macrophages, which ensures the elimination of necrotic tissue and control of infection. During this period, pro-inflammatory and regulatory mediators are activated [6]. The proliferative phase begins 4–7 days after injury and includes active angiogenesis, fibroblast proliferation, extracellular matrix synthesis, and re-epithelialization of the wound surface with the formation of granulation tissue [7,8]. The remodeling phase can last from several weeks to a year and is characterized by reorganization of the collagen matrix, the transition from type III to type I collagen, reduction in vascular density, and scar tissue formation [9], as schematically illustrated in Figure 1.

Despite the detailed understanding of the individual stages of wound healing, the problem of objective assessment of skin restoration following burn injuries—where necrosis, inflammation, and regeneration overlap—remains insufficiently developed. Imaging of burn wounds is a critically important component of clinical practice, as the choice of conservative or surgical management, healing prognosis, and the risk of patient disability all depend on accurate assessment of burn depth and area. Traditional clinical evaluation, based on determining the depth and extent of injury, remains largely subjective. The differential diagnosis between partial-thickness and full-thickness burns is particularly challenging, as an incorrect decision can lead to either prolonged non-healing of the wound or to septic complications. Consequently, there is a growing need for imaging techniques capable of simultaneously providing high spatial resolution and functional informativeness across different levels of tissue organization—from macroscopic to cellular. The primary objective of this review is to provide a comprehensive and critical analysis of the current state of imaging modalities for burn wounds. We specifically compare the principles, advantages, limitations, and clinical applicability of light and electron microscopy, fluorescence imaging (wide-field, confocal, FLIM), as well as tomographic and mass spectrometry imaging methods in the context of burn injury assessment and regeneration monitoring.

## 2. Light and Electron Microscopy

### 2.1. Light Microscopy and Histological Staining

**Figure 2:**
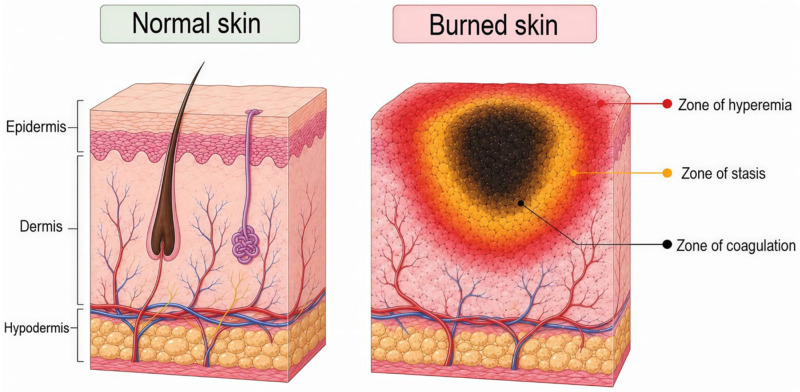
Normal skin and skin with burn zones. Generated with ChatGPT (OpenAI, GPT-5 with image generation capabilities) and manually edited by the authors.

Light microscopy with histological staining represents a fundamental method for studying the morphological changes that occur in various pathological conditions. This approach allows high precision identification of pathological tissue architecture, which is critical for assessing the depth of the injured area and selecting the appropriate therapeutic strategy [8]. Several types of histological stains exist, among which the most common and informative for burn diagnostics are hematoxylin and eosin (H&E) staining and Masson’s trichrome staining. H&E staining enables detailed visualization of the burn zones first described by Jackson (1953) [10]. According to this concept, three pathophysiological zones are distinguished in the burn area: the zone of coagulation with irreversible tissue damage, the zone of stasis where secondary deepening of the injury may occur under unfavorable conditions, and the zone of hyperemia with minimal disruption of tissue structure [10]. Figure 2 schematically shows histological sections of normal skin and a burn wound with visualization of these three zones.

At the microscopic level, the zone of coagulation is characterized by the presence of homogeneously stained necrotic tissue with loss of cellular detail, absence of normal dermal and epidermal structure due to protein denaturation and coagulation under high temperatures, and vascular occlusion. The zone of stasis appears as an area with marked edema and cellular infiltration, where tissue structures and vascular elements are still preserved, but there are a high microcirculatory disturbance and signs of ischemic cell damage. The zone of hyperemia is characterized by moderate vasodilation, a pronounced inflammatory response, and edema, without substantial disruption of tissue architecture [11,12].

However, despite its informativeness, H&E staining does not always provide an unambiguous indication of the level of fibrotic changes in tissues, especially in the later stages of burn regeneration. To address this issue, Masson’s trichrome staining is used. This method has become widespread due to its ability to differentiate connective tissue elements from other tissue structures [13]. Interpretation of images obtained using Masson’s staining relies on the clear color-based differentiation of tissue components: collagen stains blue or green, cytoplasm stains red, and nuclei stain black. In the early stages of regeneration, immature type III collagen predominates, with a less organized fiber arrangement, whereas in the later stages of healing it is replaced by mature type I collagen, characterized by a dense and ordered fiber structure [14]. Trichrome staining effectively visualizes these morphological changes and provides an accurate assessment of the degree of tissue remodeling, which is critically important for predicting functional skin regeneration after burn injuries [15].

Thus, histological staining methods serve as complementary tools for the morphological assessment of burn injuries, enabling comprehensive analysis of tissue architecture and the dynamics of regeneration processes. Hematoxylin and eosin staining provides rapid stratification of the zones of coagulation, stasis, and hyperemia, whereas Masson’s trichrome staining is particularly informative in the later stages of healing, allowing evaluation of extracellular matrix remodeling and the degree of scar maturation [13,14]. At the same time, the method remains invasive, requiring biopsy and labor-intensive sample preparation, which limits its use for dynamic in vivo monitoring and rapid clinical assessment of wound status. Consequently, despite its status as the morphological gold standard, light microscopy is primarily used to verify injury depth, assess reparative processes in fixed samples, and in preclinical studies, underscoring the need for molecularly specific and non-invasive imaging techniques.

### 2.2. Immunohistochemical Analysis

Immunohistochemistry (IHC) is a morphological method that allows for the assessment of the expression of molecular markers of inflammation, proliferation, angiogenesis, epidermal regeneration, and extracellular matrix remodeling in burn tissue [16]. The method is based on the highly specific interaction between antibodies and antigens, the latter being marker proteins whose changing levels enable tracking of the burn wound microenvironment dynamics at different stages. This selectivity is a hallmark of IHC, distinguishing it from classical histology, which visualizes general tissue structures [17].

In experimental models, IHC is used to analyze early markers of inflammation and regeneration following burn injury. The initial inflammatory phase is accompanied by increased expression of pro-inflammatory cytokines and marked infiltration of the wound bed by macrophages. However, burn wounds are characterized by persistent, protracted inflammation, which disrupts tissue architecture and reduces the functional properties of the healed area. In this context, suppression of the local inflammatory response is a promising strategy. In particular, neutralization of tumor necrosis factor alpha (TNF-α) leads to a reduction in the number of CD68^+^ macrophages in the injured area, as visualized by IHC. Thus, IHC enables an objective assessment of the cellular composition of the infiltrate and the efficacy of therapeutic intervention [15].

IHC also allows investigation of early changes in vascular activation and the mechanisms of leukocyte recruitment to the injured area. In particular, expression of ICAM 1 (intercellular adhesion molecule 1) on endothelium and keratinocytes is a critical factor in normal wound healing, and its level correlates with the completeness of epithelialization and the formation of granulation tissue. This makes ICAM 1 a potential early IHC marker of vascular and endothelial activation in the burn zone [18,19].

The transition to the proliferative phase is accompanied by activation of angiogenesis, which is routinely assessed by IHC using markers such as vascular endothelial growth factor (VEGF) and CD31. VEGF is a key regulator of angiogenesis required for effective repair, and the density of CD31^+^ microvessels increases in granulation tissue [20,21]. In a rat model of deep burn, local application of granulocyte–macrophage colony-stimulating factor (GM–CSF) was shown to accelerate angiogenesis: IHC revealed elevated VEGF levels and a shift in the angiopoietin-1/angiopoietin-2/Tie-2 balance. Double immunolabeling for CD31/Ki-67 demonstrated an increased proportion of proliferating endothelial cells at early time points, followed by increased pericyte coverage of the vessel (CD31/alpha-smooth muscle actin (α-SMA)) and vessel maturation, illustrating the ability of IHC to detect both early and late vascular changes [22].

Researchers pay particular attention to markers of cell proliferation, notably Ki-67, which are used for objective assessment of epithelialization. After burn injury, the number of Ki-67^+^ stained cells in the basal layer of the epidermis increases at the wound margins: this has been demonstrated both in a human skin explant burn model, with a proliferation peak at days eight and nine, and in mouse burn models, where an increase in Ki-67^+^ keratinocytes was observed on day seven compared with controls [23,24]. IHC is also used to evaluate fibrotic changes and the remodeling potential of the dermal layer. In the later stages of healing, staining for type I collagen and α-SMA allows assessment of extracellular matrix maturity and the degree of myofibroblast development—key cells responsible for wound contraction, collagen production, and scarring [25,26]. An excess of myofibroblasts is reliably associated with the formation of hypertrophic scars and keloids [27]. Additionally, IHC is used to analyze markers related to the regulation of fibrosis and inflammation, such as transforming growth factor beta 1 (TGF-β1) and matrix metalloproteinase 9 (MMP-9). TGF-β1 is a key inducer of collagen synthesis and fibroblast activation, whereas MMP-9, along with other metalloproteinases, participates in extracellular matrix remodeling [28,29]. Collectively, this panel of markers allows tracking of the transition from the active remodeling phase to scar stabilization, assessment of the balance between collagen synthesis and degradation, and identification of points at which the physiological process starts to shift towards pathological fibrosis.

Overall, IHC is a key method for molecularly specific assessment of burn wound status, enabling visualization and quantitative analysis of inflammatory, angiogenic, and fibrotic processes [30]. At the same time, IHC requires invasive biopsy sampling and scrupulous sample preparation, precluding dynamic in vivo monitoring. Additional limitations include protocol variability, partially subjective interpretation of results, and high cost, which can reduce reproducibility and hinder data standardization [31,32]. Consequently, the method is optimal for preclinical studies and molecular verification of regeneration processes, but is limited in routine clinical practice.

**Figure 3:**
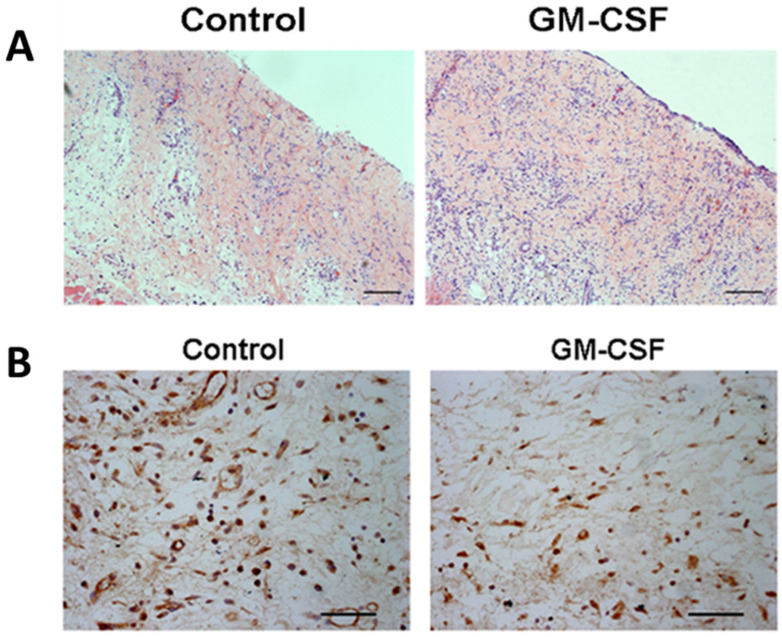
Representative images of skin tissue obtained using hematoxylin and eosin staining (**A**) and immunohistochemistry (**B**). Scale bar = 50 mm. Reproduced under terms of the CC-BY license [22].

Representative images acquired by hematoxylin and eosin staining and by immunohistochemistry (IHC) using a panel of antibodies against rat Ang-1 are presented in Figure 3.

### 2.3. Electron Microscopy (SEM, TEM)

Electron microscopy is a powerful experimental tool for studying morphological and ultrastructural changes in the skin after burns; however, due to its technical complexity and labor-intensive sample preparation, it is not a routine clinical method for diagnosing burn injuries. Depending on the mode, electron microscopy either provides three-dimensional images of tissue surfaces (scanning electron microscopy (SEM)) or enables detailed visualization of intracellular structures (transmission electron microscopy (TEM)).

Scanning electron microscopy is based on scanning the sample surface with a focused electron beam [33]. The method generates images with high depth of field and a three-dimensional perception of tissue microtopography [34]. These properties make SEM a valuable tool for studying architectural changes in the skin after burns with submicron resolution. SEM allows visualization of the structural integrity of collagen fibers, the state of cells, and components of the extracellular matrix [35]. In addition to assessing morphological changes in the tissue itself, SEM is also important for analyzing secondary processes that complicate burn wounds, in particular bacterial colonization and the interaction of the wound bed with synthetic matrices. Burn injury exposes the wound surface, making it vulnerable to bacterial colonization and creating conditions for biofilm formation by opportunistic bacteria such as *Pseudomonas aeruginosa* and *Staphylococcus aureus*. Persistence of these microbial communities significantly increases the risk of chronic infection. SEM enables visualization of the three-dimensional structure of biofilms, assessment of bacterial mass density, and morphology of microbial associates [36,37]. These studies are important for understanding the structural organization of biofilms and can be used to develop antibacterial therapy strategies. Beyond microbial community analysis, SEM is widely used to evaluate local burn therapy agents, namely skin equivalents, hydrogels, and biodegradable matrices, to study their interaction with adjacent tissue. The method allows assessment of material porosity and microtopography, cell attachment, and early stages of extracellular matrix formation. For layered nanofiber sponges, it has been shown that their three-dimensional architecture improves interaction with blood cells and accelerates hemostasis. SEM confirmed the optimal architecture of the sponges, and in a full-thickness skin defect model, such architecture was associated with faster healing and reduced scarring [38]. For clay-reinforced neomycin-loaded nanofibers, SEM confirmed uniformity and porosity. These structural characteristics correlated with accelerated wound closure and improved collagen organization in a rabbit burn model [39]. Similar results were obtained for a fibroblast growth factor 2 (FGF-2)-collagen-containing hydrogel in rats: SEM visualized the restored epidermal layer and a more organized dermal structure, consistent with histological findings [40].

Transmission electron microscopy complements SEM by enabling visualization of intracellular structures and organelles at nanometer resolution. TEM allows the study of membranes, mitochondrial cristae, nuclear structures, the cytoskeleton, intercellular junctions, and the extracellular matrix. A comparative characterization of the two methods is presented in Table 1.

In preclinical burn models, TEM is used for the morphometry of collagen fibrils. The method assesses the distribution of fibril diameters across dermal layers depending on the stage of healing and scarring, reflecting changes in fibrillogenesis and extracellular matrix organization. To evaluate cell proliferation, TEM is often combined with ^3^H thymidine autoradiography or IHC, enabling correlation of ultrastructural features of cells with their proliferative activity and the dynamics of granulation tissue formation [41]. The method is also used to assess the restoration of the basement membrane and the contact between the newly formed epithelium and granulation tissue, serving as an important morphometric confirmation of functional conclusions about the healing process. In cellular energetics research, TEM visualizes mitochondrial changes and permits their stereological analysis. For instance, severe thermal injury in rats led to a decrease in mitochondrial count and disruption of cristae in cardiac muscle [42], while in mouse skeletal muscle, mitochondria became enlarged and lost their cristae—alterations that were reversible with pharmacological intervention [43]. In preclinical studies, these parameters serve as sensitive indicators of energy deficit, systemic catabolism, and therapeutic efficacy.

In fundamental research, SEM and TEM are often combined for comprehensive tissue evaluation. The combination of these methods has enabled detailed analysis of ultrastructural changes in the skin following thermal injury. TEM reveals critical damage to organelles, chromatin condensation, and disruption of intercellular junctions, allowing differentiation between zones of irreversible necrosis and areas of potential regeneration. SEM visualizes surface topography, coagulation, and fragmentation of collagen fibers, and microvascular thrombosis. The combined use of the two methods demonstrates a correlation between the degree of type I collagen depolymerization, basement membrane integrity, and burn depth, confirming their value for objective assessment of injury severity [44].

**Figure 4:**
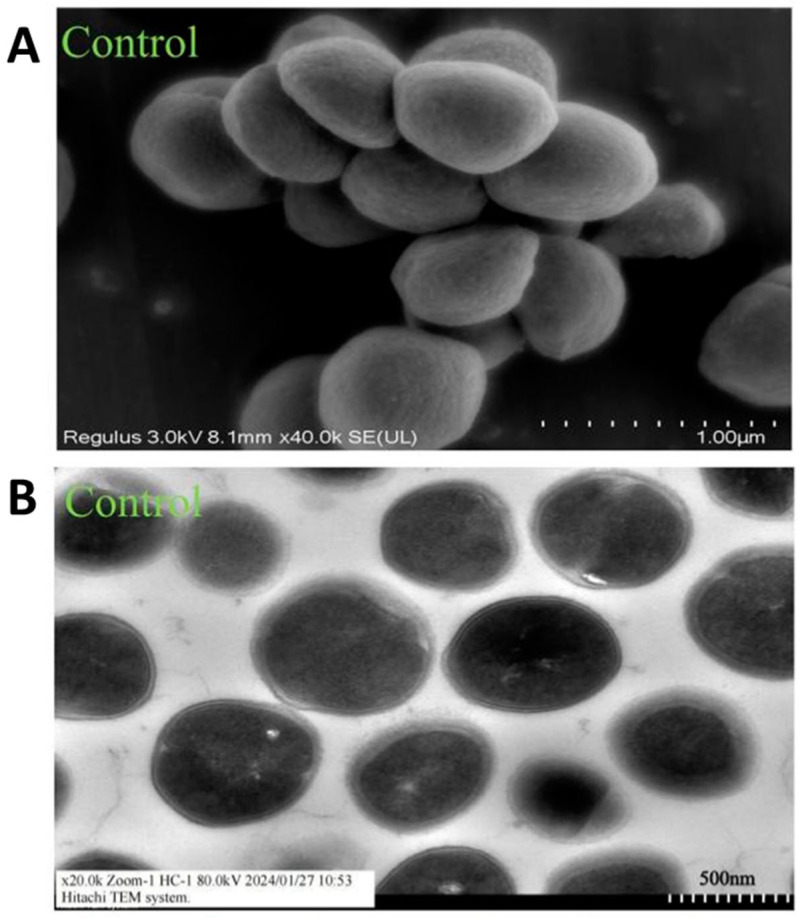
Representative images of bacterial biofilms obtained using scanning electron microscopy (**A**) and transmission electron microscopy (**B**). Reproduced under terms of the CC-BY license [45].

Figure 4 shows representative images of bacterial biofilms obtained using scanning electron microscopy (SEM, panel A) and transmission electron microscopy (SEM, panel B).

Thus, electron microscopy provides a detailed examination of burn wounds, enabling researchers to analyze the condition of cellular organelles, intercellular junctions, and extracellular matrix organization, as well as differentiate between reversible and irreversible tissue damage [46]. Electron microscopy remains an important method for fundamental research into the mechanisms of cellular injury and remodeling. However, its use is limited by the need for invasive tissue sampling, complex and time-consuming sample preparation, and the inability to obtain functional information from living cells. Consequently, electron microscopy serves as a high-precision verification method, but its role is largely confined to experimental and preclinical tasks.

## 3. Fluorescence Microscopy (Optical Imaging of Endogenous/Exogenous Fluorescence)

### 3.1. Wide-Field Fluorescence Imaging

Wide-field fluorescence imaging (WFFI) is an optical method in which fluorescence excitation and signal detection occur simultaneously over the entire area of interest, allowing two-dimensional image acquisition without scanning. For superficial skin structures and open burn wounds, the method is suitable due to its high temporal throughput and relative instrumental simplicity, although depth resolution is limited by out-of-focus light contribution [47,48,49].

*Indocyanine Green Angiography*. The most advanced application of WFFI in burns is indocyanine green (ICG) angiography. After intravenous administration of ICG, dynamic near-infrared fluorescence recording is performed over the entire wound bed. Pilot clinical studies have shown that the ratio of fluorescence intensity in the burn area to that in uninjured skin correlates with injury depth: higher values are characteristic of superficial burns, whereas reduced signal is associated with deep injuries [50]. Not only amplitude parameters but also ICG accumulation kinetics have diagnostic value: the rate of fluorescence signal increase and the shape of perfusion curves reflect microcirculatory status and allow more accurate burn depth differentiation [51]. In vivo models in pigs and rodents have confirmed the feasibility of monitoring the dynamics in the zone of stasis and early necrosis progression, which is important for optimizing the timing of surgical intervention [52,53]. In a prospective, multicenter, triple-blinded study by Wongkietkachorn et al. (2019), including 30 burn sites in stable patients with indeterminate-depth burns assessed within one week after injury, ICG angiography achieved an accuracy of 100% for burn depth stratification using histology as the reference standard, compared with 50% for conventional clinical visual assessment [54]. The study employed a standardized protocol involving intravenous administration of indocyanine green (0.2 mg/kg) followed by near-infrared imaging. Although these findings demonstrate the considerable diagnostic potential of ICG angiography, they should be interpreted with caution because of the relatively small sample size, the specific patient cohort studied, and variability in imaging protocols across different studies. Beyond diagnostic stratification, intraoperative application of ICG angiography enables precise delineation of tissue requiring excision, thereby reducing the risk of both insufficient and excessive tissue removal and potentially improving healing outcomes [55]. In addition, reduced initial perfusion and delayed fluorescence dynamics have been associated with the progression of tissue necrosis, whereas preserved vascular responses correlate with spontaneous re-epithelialization.

*Protoporphyrin IX-Based Analysis*. Wide-field recording of endogenous protoporphyrin IX (PpIX) fluorescence, particularly in combination with ICG angiography, enables differentiation of necrosis and hypoxia zones (via PpIX) from inflammation zones (via ICG) in burn wounds. It is important to distinguish two types of protoporphyrin IX signal: prompt fluorescence, which reflects PpIX accumulation in non-viable tissue and bacterial biofilms, and delayed fluorescence, which assesses the degree of tissue hypoxia [56]. PpIX accumulation is associated with non-viable tissue areas, indirectly indicating reduced regenerative potential [57]. PpIX fluorescence can also serve as a marker for metabolic stress and be used to evaluate the effectiveness of photodynamic therapy [58,59]; however, its prognostic value regarding pathological scarring requires further investigation.

*Assessment of Bacterial Dissemination*. Excitation of bacterial porphyrins generates a wide-field image in which areas of clinically significant contamination (≈10^4^ CFU/g and above) appear as characteristic fluorescent foci, mapping microbial “hot spots” across the entire wound surface [60,61]. The use of wide-field fluorescence increases the detectability of bacterial load, facilitates targeted sampling, and helps optimize the extent of surgical debridement [61]. Incorporating bacterial fluorescence imaging into wound management protocols enables earlier initiation of targeted antimicrobial therapy and reduces the rate of complications [62,63,64].

*Wide-Field Fluorescence Imaging in Tissue Engineering for Skin Regeneration Analysis.* WFFI has become a standard tool for monitoring cell behavior in three-dimensional constructs. In humanized skin models with fluorescently labeled keratinocytes, the method provides a quantitative assessment of epithelial migration, basement membrane remodeling, and cellular responses to growth factors [65]. In bioengineered skin equivalents, WFFI is used to evaluate structural protein expression, basement membrane organization, and cell viability [66]. Modern full-thickness skin equivalents derived from telomerase reverse transcriptase (TERT)-immortalized cell lines allow for the modeling of thermal injury in vitro and analysis of re-epithelialization dynamics using immunofluorescence detection [67].

*Wide-Field Fluorescence Imaging in Assessment of Extracellular Matrix Remodeling*. WFFI, particularly in the UV range, exploits the endogenous fluorescence of collagen, elastin, and aromatic amino acids to assess the structural state of the dermis and indirectly monitor epithelialization. In an experimental human skin organotypic culture, it was demonstrated that zones of exposed dermis can be identified by collagen signal, and the re-epithelialization front can be determined by epidermal fluorescence [68,69]. In addition to endogenous fluorescence, exogenous contrast agents can be used to visualize damaged collagen. Fluorescently labeled collagen mimetic peptides (cyanine dye 5-conjugated to collagen-mimetic peptide (Cy5-CMP)) selectively bind to denatured collagen, generating contrast maps of injury [70].

**Figure 5:**
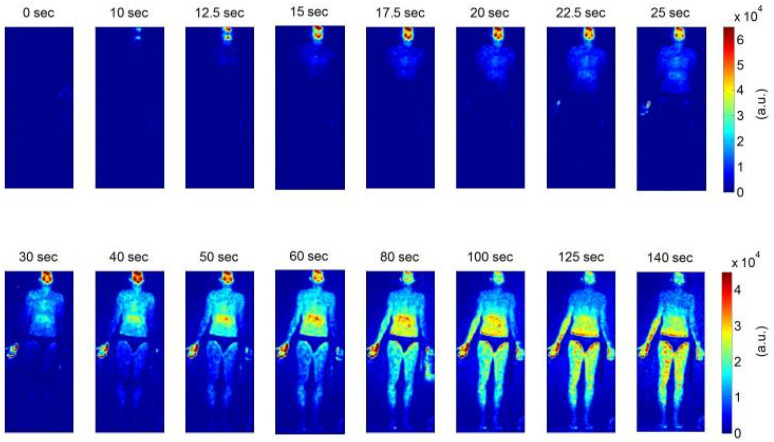
Representative wide-field near-infrared reflectance images. Reproduced under terms of the CC-BY license [71].

As an example of wide-field imaging, Figure 5 presents a sequence of frames demonstrating the propagation of ICG through the skin.

Thus, WFFI is a high-throughput tool for assessing perfusion, bacterial load, and regeneration dynamics. The method provides a large field of view and high acquisition speed, which is important for burn depth stratification and intraoperative mapping. Limitations of the method include the lack of optical sectioning and limited penetration depth, which reduce the accuracy of analyzing deep structures. For detailed morphofunctional analysis, WFFI requires a combination with methods of higher spatial resolution.

### 3.2. Confocal Fluorescence Microscopy

Confocal fluorescence microscopy (CFM) is an optical laser-scanning method that enables acquisition of thin optical sections of tissues with high resolution and contrast. The principle of CFM involves scanning a sample point by point with a focused laser beam and the use of a confocal aperture (pinhole) to eliminate out-of-focus light and provide optical sectioning [72]. Unlike wide-field imaging, CFM allows the generation of a series of optical sections (Z stacks) and three-dimensional reconstructions of tissues. This is important for quantitatively assessing morphological and functional changes in the skin at the cellular level [73]. The method can work with both endogenous fluorophores (flavins, nicotinamide adenine dinucleotide (NADH), collagen, and elastin) and exogenous labels (fluorophores, labeled antibodies, and fluorescent probes).

*Imaging of Vascular Pathology*. One of the key applications of CFM in thermal injury is the visualization of early vascular pathology. In mouse burn models with intravenous administration of high-molecular-weight fluorescein isothiocyanate (FITC)-dextran, CFM enabled visualization of microcirculatory changes in the injured area and perifocal regions, differentiating the zones of coagulation (absence of perfusion), stasis (slowed blood flow), and hyperemia (enhanced perfusion). Vessel maps correlated with data from vascular corrosion casts obtained four hours after burn [74]. In addition to the assessment of vascular changes, CFM with indocyanine green improves the contrast ratio between epidermal and dermal structures, enhancing the distinction between skin layers and appendages, thereby providing a basis for more precise delineation of burn wound boundaries [75].

*Burn Depth Stratification*. In a clinical study of 14 patients, CFM demonstrated high diagnostic value for differentiating between superficial, superficial partial-thickness, and deep partial-thickness burns as early as 24 h after injury. In superficial burns, the size of cells in the stratum granulosum increases, the basal layer thickens, and the number of perfused dermal papillae increases. In superficial partial-thickness burns, the stratum granulosum is destroyed, the basal layer is partially disrupted, and the number of perfused papillae decreases. Deep partial-thickness burns are characterized by complete destruction of the basal layer and absence of perfused papillae. Thus, the method enables early stratification of burns by injury depth, accelerating the selection of appropriate therapeutic intervention [76].

*Analysis of Re-epithelialization, Angiogenesis, and Matrix Remodeling*. CFM enables tracking of key events in skin restoration after burns, most notably the dynamics of re-epithelialization. In a study using keratin-5 promoter-driving green fluorescent protein (K5-GFP) transgenic mice, CFM captured spatiotemporal changes in keratinocyte activity. GFP intensity increased at the leading edge of keratinocyte migration, reflecting enhanced proliferation. Maximal values were observed on days 12–15, after which the signal decreased as re-epithelialization was completed. GFP expression levels correlated with immunohistochemistry results for keratin-5, a marker of basal keratinocytes [77]. CFM is also widely used to assess angiogenesis and extracellular matrix remodeling in histological sections. In a deep-burn mouse model, a pro-angiogenic collagen implant significantly increased the number of CD31^+^/α-SMA^+^ vessels on days 14–35, as shown by confocal imaging, accompanied by accelerated tissue repair [78]. In a rat full-thickness burn model, CFM was used to verify skin wound reinnervation: co-localization analysis of growth-associated protein-43 (GAP-43), protein gene product 9.5 (PGP9.5), calcitonin gene-related peptide (CGRP), and tyrosine hydroxylase markers allowed detailed characterization of the regeneration dynamics of different nerve fiber types [79].

**Figure 6:**
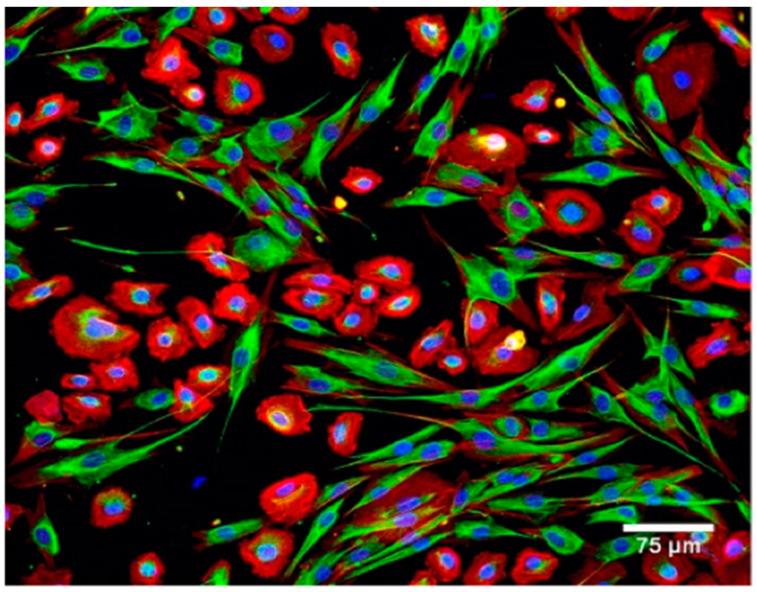
Representative confocal microscopy image of a co-culture of human skin fibroblasts and keratinocytes. Reproduced under terms of the CC-BY license [80].

Figure 6 shows a representative image obtained using confocal laser scanning microscopy. The image shows a co-culture of human skin fibroblasts and keratinocytes, with fibroblasts showing blue fluorescence of nuclei, red fluorescence of actin filaments, and green fluorescence of vimentin filaments, and keratinocytes showing blue fluorescence of nuclei and red fluorescence of actin filaments.

Despite its high sensitivity and spatial resolution, CFM has several limitations. These include limited penetration depth under one-photon excitation, signal attenuation beneath eschar and necrotic tissue, dependence on exogenous dyes, risks of phototoxicity, a narrow field of view, high equipment costs, and training requirements. Overall, CFM provides high-contrast optical sectioning and enables analysis of microcirculation, re-epithelialization, extracellular matrix remodeling, and microbial biofilms at the cellular level. The method is particularly valuable for early stratification of burn depth and functional monitoring of regeneration in vivo and ex vivo. However, limited penetration depth and a small field of view make CFM optimal for detailed local analysis but not a replacement for wide-field imaging methods.

### 3.3. Fluorescence Lifetime Imaging Microscopy (FLIM)

Fluorescence lifetime imaging microscopy (FLIM) is an imaging method based on differences in the exponential decay of sample fluorescence. The image is formed not by intensity but by the fluorescence lifetime of the fluorophore—the interval between molecule excitation and photon emission. Fluorescence lifetime is sensitive to changes in metabolism, pH, viscosity, oxygen levels, and intermolecular interactions, enabling the detection of changes not captured by standard fluorescence microscopy. FLIM distinguishes fluorophores with the same excitation wavelength but different lifetimes and, in some cases, works without labels by exploiting the sample’s endogenous fluorescence [81].

Cells contain endogenous fluorophores that participate in redox reactions and play a key role in metabolism. The main energy-producing pathways are oxidative phosphorylation (in mitochondria, oxygen-dependent) and glycolysis (oxygen-independent) [82]. The coenzyme NAD is involved in all these processes and serves as a reliable metabolic indicator [83]. NAD exists in cells in its reduced (NADH), oxidized (NAD^+^), and phosphorylated (NADPH or NADP^+^) forms. Among all redox cofactors in the cell, only NADH, NADPH, and flavin adenine dinucleotide (FAD) exhibit fluorescence signals, with NADH showing the highest intracellular fluorescence intensity [84,85]. NADH exists in a free form (cytosol, glycolysis) and a protein-bound form (mitochondria, oxidative phosphorylation) [86,87]. These states differ in fluorescence lifetime, allowing FLIM to monitor the balance between glycolysis and oxidative phosphorylation [88,89].

In the context of burn research, such studies are currently very limited, but FLIM appears highly promising. The method can non-invasively assess metabolic disturbances in the skin after thermal injury, distinguishing zones of necrosis, persistent ischemia, and regeneration based on the lifetime of endogenous fluorophores (primarily NAD(P)H), which may aid in early burn depth diagnosis and treatment selection without biopsies or contrast agents. For example, Malak et al. (2025) demonstrated that FLIM can non-invasively track keratinocyte differentiation—a key process in epidermal restoration after burn [90]. Upon induction of differentiation, increased expression of keratin 1 and keratin 10 markers was observed, along with a metabolic shift from glycolysis to oxidative phosphorylation. The method allowed monitoring of differentiation over 96 h, and FLIM data analysis correlated well with differentiation time. Thus, FLIM can provide useful parameters for monitoring keratinocyte status, which is important for assessing skin regeneration after burn injury [90].

**Figure 7:**
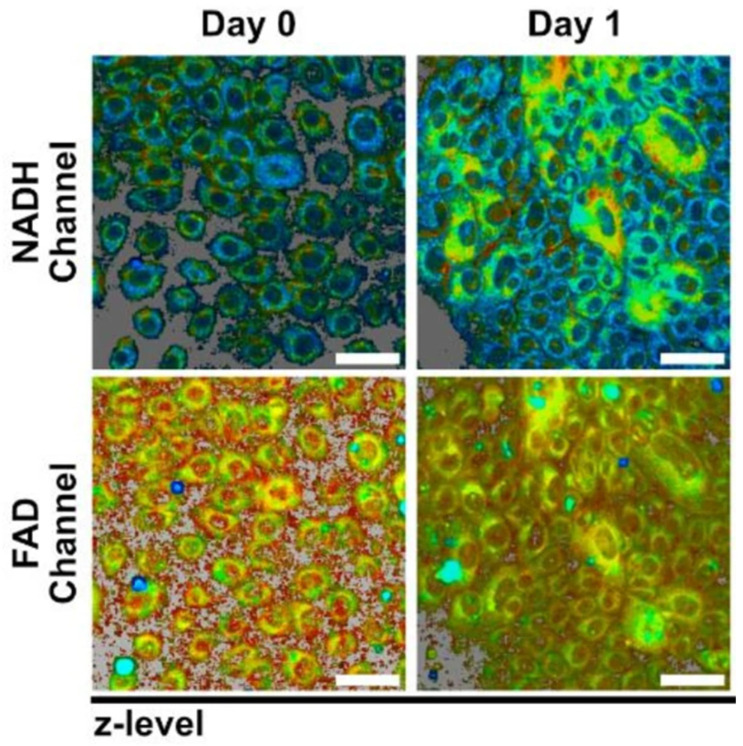
Representative FLIM images of live human keratinocytes cultured in a high-calcium medium. Scale bar: 50 μm. Reproduced under terms of the CC-BY license [90].

Figure 7 presents FLIM images of live human keratinocytes cultured in a high-calcium medium for four days, with colors indicating the pixelwise mean fluorescence lifetime (τm).

In another study, FLIM was used for the first time to characterize macrophage metabolism directly in the wound microenvironment, including following thermal injury. It was shown that inhibition of glycolysis reduced the mean fluorescence lifetime of NAD(P)H and made the redox state of macrophages more oxidized. TNF-α^+^-macrophages exhibited a lower mean NAD(P)H fluorescence lifetime and a more oxidized state compared with TNFα^−^-cells. Both infection and thermal injury induced the appearance of a macrophage population with a more oxidized redox state in wound tissues. As healing progressed, FLIM analysis revealed a gradual shift toward a more reduced state. Thus, FLIM is sensitive to the dynamics of intracellular macrophage metabolism in tissues and can be used to assess the regulation of macrophage metabolism during tissue injury and repair, including burn trauma [91].

In burn research, FLIM may be particularly useful when combined with second harmonic generation (SHG), as this approach allows simultaneous assessment of cellular metabolism and collagen matrix status. In a study by Deka et al. (2013) [92] using a rat wound-healing model, the combination of FLIM and SHG revealed increased metabolic activity during the first week of healing, with a gradual decline after day eight. SHG also detected collagen degradation during the inflammatory phase, and the onset of collagen regeneration starting from day five, followed by a progressive accumulation in scar tissue. Thus, the combination of FLIM and SHG enables non-invasive tracking of both cellular metabolic changes and extracellular matrix remodeling during wound healing, which holds promise for burn wound diagnostics [92]. In another study, the FLIM SHG combination allowed simultaneous assessment of two key processes during wound healing following interleukin-12 administration: FLIM recorded a marked increase in cellular metabolic activity in the wound at early time points, while SHG revealed less pronounced changes in collagen structure compared with controls [93]. Furthermore, FLIM–SHG provides information on the structure of the dermal papillary layer, including collagen and elastin distribution, and can characterize capillaries based on the fluorescence lifetimes of red blood cells and blood plasma, which is relevant for diagnosing connective tissue and vascular disorders in burns and other skin injuries [94]. FLIM can also non-invasively assess cellular energy metabolism in dermal equivalents, which is especially important for tissue engineering and transplantation. This allows monitoring of the quality of cellular components in living skin substitutes before and after grafting without the need for external labels [95].

Despite all its advantages, FLIM currently remains primarily an experimental method, and its clinical application in burns is associated with several limitations. The main ones are shallow penetration depth (hundreds of micrometers, even under two-photon excitation), which precludes assessment of the deep dermis and subcutaneous tissue in severe burns; complex data analysis requiring calibration and standardization, which hinders result reproducibility; and high equipment costs. Furthermore, widespread clinical implementation and validation of FLIM techniques are hampered by a lack of cooperation between optical system manufacturers, clinical partners, and clinical equipment developers [96,97].

## 4. Tomographic and Mass Spectrometry Imaging Methods

### 4.1. MRI and Ultrasound Imaging

Magnetic resonance imaging (MRI) is highly sensitive to edema and changes in soft tissue, but its application in acute burns is limited by high cost, long examination time (20–40 min), motion artifacts, and limited availability in the acute trauma setting. In routine practice, MRI is not used for burn depth stratification; however, it is employed for research purposes, as well as when deep spread of injury to muscles and fascia is suspected or to evaluate complications. In preclinical studies, MRI with T2-weighted and post-contrast sequences can differentiate viable tissue from necrotic zones and track the distribution of labeled cells [98,99,100].

Unlike MRI, high-frequency ultrasound (20–50 MHz) is more accessible and rapid and can be used at the patient’s bedside. It provides submillimeter resolution (50–100 μm), enabling visualization of the epidermis and dermis, measurement of edema thickness, and detection of structural changes [101]. For burn wound assessment, three different but often complementary ultrasound techniques are effectively used: tissue harmonic imaging, elastography, and Doppler mode. Tissue harmonic imaging provides high-resolution images and clearly visualizes burn depth, skin layers, and vascular status. Elastography, in turn, is a separate functional mode that assesses the mechanical properties of tissues, namely their stiffness or elasticity, by measuring deformation under external stress. The use of these two modes allows simultaneous assessment of the anatomical structure of the burn wound and the biomechanical characteristics of the forming tissue, which is critically important for monitoring the healing process [102,103]. Doppler mode enables assessment of blood flow in vessels ranging from tens to hundreds of micrometers in diameter. However, it does not directly visualize true capillary microcirculation. Despite this, assessment of dermal microcirculation is crucial for determining burn depth. The state of microvessels is a sensitive indicator of tissue damage, making it more reliable than visual assessment. In practice, the thickness of the non-perfused layer (the tissue area without detectable blood flow measured from the wound surface to the first identified vessel) can correlate with necrosis depth. Generally, an increase in this thickness is associated with deeper injury. Therefore, Doppler ultrasound can be used to estimate viable tissue levels prior to tangential excision, helping to reduce the risk of over resecting the preserved dermal layer [104,105].

**Figure 8:**
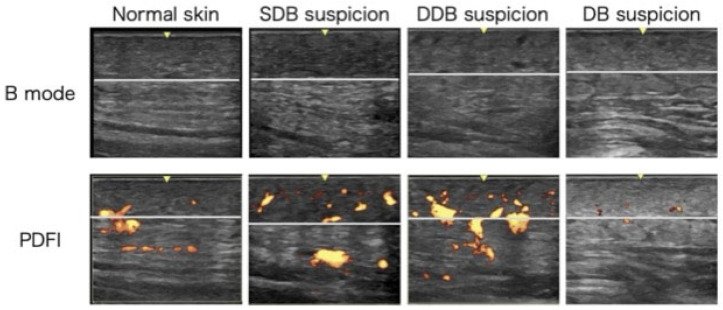
Representative high-frequency ultrasound images of burn wounds. Reproduced under terms of the CC-BY license [105].

Figure 8 presents representative ultrasound images used for burn wound assessment, including superficial dermal burns (SDBs), deep dermal burns (DDBs), and deep burns (DBs). 

Thus, MRI and ultrasound are complementary non-invasive methods for assessing burn depth, microcirculation, and scarring. Comparison of the methods is presented in Table 2. MRI is sensitive to soft tissue changes and deep necrosis but is expensive and less accessible. High-frequency ultrasound provides bedside real-time imaging of superficial structures and blood flow. Their combination improves the accuracy of burn stratification; however, both methods are limited when it comes to analyzing cellular and molecular changes.

### 4.2. Mass Spectrometry Imaging

Mass spectrometry imaging (MSI) is an analytical method that allows for the label-free spatial mapping of a wide range of molecules, including lipids, proteins, metabolites, and drugs, directly in tissue samples. This method eliminates the need for the use of antibodies or exogenous labeling, making it a valuable tool for researchers [106]. By providing untargeted, high-content molecular information, MSI adds a crucial molecular dimension to conventional histopathology and is particularly valuable for studying complex processes such as burn injury and subsequent tissue regeneration. Depending on the ionization technique (e.g., matrix-assisted laser desorption/ionization (MALDI) or desorption electrospray ionization (DESI)), spatial resolution ranges from a few to tens of micrometers, allowing correlation of molecular profiles with distinct morphological zones of the burn wound—epidermis, dermis, and the regions of necrosis, stasis, and hyperemia—when aligned with histology [107,108]. In the context of burn wounds, MSI is of particular interest because it can reveal phase-specific molecular changes underlying skin injury and regeneration, including protein and metabolite dynamics that correlate with histological tissue changes across the inflammatory, proliferative, and remodeling phases [109]. This points to the method’s potential for refining burn depth assessment and evaluating regenerative processes. Furthermore, spatial metabolomics of hypertrophic scar tissue identified over 1600 metabolites, revealing significant metabolic remodeling and suggesting novel therapeutic targets for scar treatment [110]. A particularly powerful application is the analysis of the extracellular matrix in fibrotic conditions, where MSI can localize collagen peptide sequences and N-glycosylation patterns to specific fibrotic regions, providing insight into collagen architecture remodeling and ECM signaling in pathological scarring [111]. In addition to endogenous molecules, MSI is used to study the distribution of drugs within burn wounds and to assess the penetration of topical agents, including analysis of their distribution across skin layers and interaction with endogenous lipids [112,113]. In experimental models, MSI has also enabled in situ identification of bacterial markers, including *Pseudomonas aeruginosa*, and spatial localization of infected areas, which may be important for studying wound infection [114]. Overall, current directions include three-dimensional MSI and its combination with proteomics, allowing reconstruction of the spatiotemporal dynamics of molecular changes in the burn wound at different stages of healing [115,116]. However, such approaches remain predominantly research oriented.

**Figure 9:**
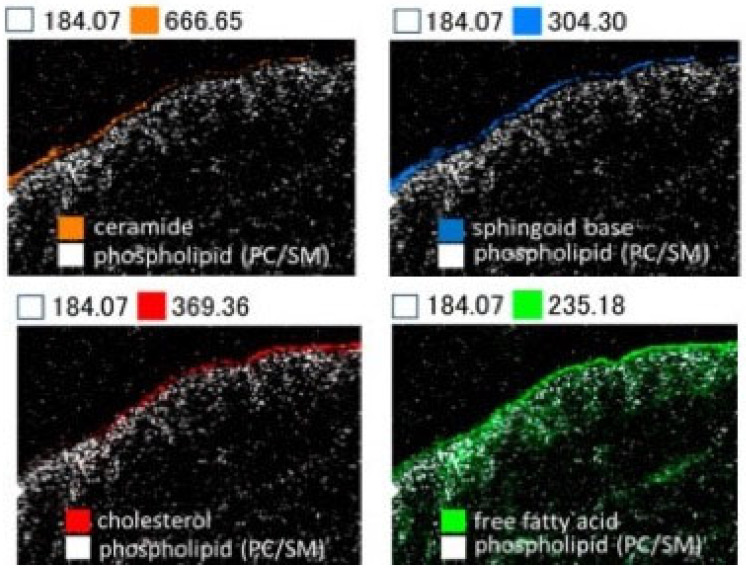
Representative images of skin tissue obtained using mass spectrometry imaging. Reproduced under terms of the CC-BY license [117].

Figure 9 presents representative images obtained using obtained using mass spectrometry imaging of skin tissue.

Despite its high information content, MSI has several limitations: the analysis is partially destructive, identification accuracy is limited, quantitative interpretation is challenging, and equipment costs are high. Furthermore, variability in sample handling (e.g., fresh-frozen vs. formalin-fixed paraffin-embedded tissues) can significantly affect the detected molecular profiles, and issues with data standardization and reproducibility across multiple centers remain major obstacles to clinical routine. Consequently, MSI is currently used primarily in fundamental and translational burn research, serving as a high-value discovery tool to elucidate molecular mechanisms of burn wound pathology and regeneration, while its clinical application remains limited by technological complexity and the need for standardization.

## 5. Comparison of Methods and Their Potential for Clinical Burn Assessment

The imaging and morphological analysis methods for burn wounds presented above differ substantially in invasiveness, spatial resolution, penetration depth, feasibility of dynamic monitoring, and clinical applicability. A comparative characterization of these methods is provided in Table 3 and Table 4. Table 3 summarizes the technical characteristics of the imaging modalities, while Table 4 outlines their translational and practical features.

Light microscopy (histology and IHC) remains the morphological gold standard: it provides cellular and subcellular resolution, enables visualization of the zones of coagulation, stasis, and hyperemia, and allows assessment of collagen remodeling and expression of molecular markers of inflammation, angiogenesis, and fibrosis. However, the method is invasive, labor-intensive, precludes dynamic in vivo monitoring, and is unsuitable for routine clinical burn depth stratification. Electron microscopy provides nanometer-scale resolution and detailed visualization of the ultrastructure of cells, organelles, and the extracellular matrix, but it is even more complex, expensive, and confined to fundamental research.

Fluorescence microscopy enables in vivo optical imaging. Wide-field fluorescence imaging is high-throughput, has a large field of view, and is the most clinically advanced: ICG angiography is successfully used for intraoperative perfusion mapping and burn depth stratification, while bacterial autofluorescence enables detection of microbial “hot spots”. Limitations include the lack of optical sectioning and limited penetration depth. Confocal microscopy provides optical sections at the cellular level, allows differentiation of superficial, partial thickness, and deep burns based on basal layer status and papillary perfusion, and enables tracking of re-epithelialization and angiogenesis. Its drawbacks are a narrow field of view, shallow depth, dependence on contrast agents, and high cost. FLIM assesses cellular metabolism without exogenous labels, allowing non-invasive distinction between zones of necrosis, ischemia, and regeneration. When combined with second harmonic generation, FLIM provides simultaneous assessment of cellular metabolism and collagen matrix status. However, the method remains primarily experimental due to shallow penetration depth, complex analysis, and high cost.

Tomographic methods occupy an important niche for in vivo assessment of deep structures. MRI has high sensitivity to soft tissue edema and necrosis and can evaluate deep layers, but is not used in routine burn practice due to high cost, long examination time, motion artifacts, and limited availability. High-frequency ultrasound, in contrast, is an accessible, rapid, bedside method with submillimeter resolution, allowing visualization of skin layers and measurement of edema and necrosis thickness. The combination of three modes—harmonic imaging (anatomy), elastography (tissue mechanical properties), and Doppler (assessment of blood flow and non-perfused layer thickness as an indicator of necrosis depth)—makes ultrasound the most balanced clinical tool for burn depth stratification and healing monitoring. The main limitation is the inability to directly visualize true capillary microcirculation. Mass spectrometry imaging occupies a unique position: it enables label-free mapping of the distribution of hundreds of molecules in tissue sections, revealing phase-specific molecular profiles of inflammation, proliferation, and remodeling, as well as identifying bacterial markers. However, the method is destructive, complex, expensive, and currently used exclusively in fundamental and translational research, with no clinical application in burns.

## 6. Conclusions

This review summarized the principal methods for imaging and morphological analysis of burn wounds, ranging from classical histology to advanced optical, tomographic, and mass spectrometry-based techniques. The clinical and experimental utility of these modalities differs substantially depending on the specific diagnostic or research objective. For acute burn depth assessment, high-frequency ultrasound appears to be the most practical bedside modality because it is rapid, accessible, and capable of visualizing skin layers and estimating necrosis depth with submillimeter resolution. For perfusion assessment and intraoperative delineation of viable tissue, wide-field fluorescence imaging with indocyanine green angiography currently represents the most clinically advanced approach due to its ability to dynamically map microcirculation and identify zones at risk of burn wound progression. For infection detection, bacterial fluorescence imaging enables rapid visualization of microbial “hot spots” across the wound surface and may improve targeted sampling and antimicrobial management. Assessment of scar maturation and extracellular matrix remodeling still relies primarily on histology and immunohistochemistry, which remain the morphological gold standard for evaluating collagen organization, myofibroblast activation, angiogenesis, and fibrosis-related markers. In contrast, mass spectrometry imaging is currently the most informative modality for preclinical molecular analysis because it enables label-free spatial mapping of metabolites, lipids, proteins, and drug distribution within burn tissue. Complementary experimental techniques such as FLIM, confocal microscopy, MRI, and electron microscopy provide additional metabolic, cellular, ultrastructural, and deep tissue information but remain predominantly research-oriented because of technical complexity, limited accessibility, or high cost. Overall, the most promising strategy for clinical burn assessment is a multimodal approach combining ultrasound and wide-field fluorescence imaging, while advanced optical and molecular methods remain essential for translational and preclinical research.

## Figures and Tables

**Table 1:** Comparison of SEM and TEM in burn wound morphology.

Parameter	SEM	TEM
Type of electron interaction	Secondary electrons from the surface	Electrons transmitted through the section
Resolution	1–10 nm	0.1–0.2 nm
Type of structure imaged	Surface microstructure	Subcellular, organelles
Requirement for ultrathin sections	Not required	Required (50–100 nm)
Area of study	Tissue microtopography, extracellular matrix	Cells, organelles, mitochondria
Suitable for surface analysis	Yes	No
Suitable for internal ultrastructure analysis	No	Yes
Application in burn studies	Surface analysis, assessment of coagulation, stasis, scar formation	Evaluation of cellular damage, mitochondria, apoptosis, synthetic activity

**Table 2:** Comparison of MRI and ultrasound in burn wound morphology.

Parameter	MRI	Ultrasound
Type of information obtained	Morphological and functional (edema, necrosis, contrast-enhanced perfusion)	Structural and vascular (skin thickness, echogenicity, Doppler perfusion)
Depth of assessment	Deep soft tissues, fascia, muscles	Primarily superficial and mid-dermal layers
Spatial resolution	Millimeter level	Down to hundreds of micrometers (at 20–50 MHz)
Assessment of microcirculation	Indirect (contrast enhancement, dynamic sequences)	Direct (power Doppler, contrast-enhanced ultrasound)
Bedside applicability	Limited	Feasible
Availability and cost	High cost, stationary equipment	More accessible and portable
Main niche in burns	Depth stratification, assessment of deep tissue viability, graft monitoring	Burn depth assessment, dermal status, scarring, and perfusion dynamics

**Table 3:** Technical characteristics of imaging modalities.

Method	Principle	Spatial Resolution	Penetration Depth	Field of View	Acquisition Time	Contrast Agents
Light microscopy	Tissue staining and morphological assessment	~0.2 µm	Superficial/section-based	Medium	Minutes–hours	Usually required
IHC	Antibody–antigen binding	~0.2 µm	Superficial/section-based	Medium	Hours	Required
SEM	Electron beam surface scanning	1–10 nm	Surface only	Small–medium	Hours	Not required
TEM	Electron transmission through ultrathin sections	<1 nm	Ultrathin sections only	Very small	Hours	Often required
WFFI	Full-field epifluorescence	~200–300 nm lateral	~100–200 µm	Large	Seconds	Usually required
CFM	Laser scanning with a pinhole	~200–300 nm lateral	~100–300 µm	Small	Seconds–minutes	Usually required
FLIM	Fluorescence lifetime mapping	~200–300 nm lateral	~100–300 µm	Small–medium	Seconds–minutes	Not always required
MRI	Proton NMR signal	~0.1–1 mm	Deep tissue	Large	Minutes	Optional
Ultrasound	Echolocation	~50–100 µm	Several cm	Large/ adjustable	Real time	Optional
MSI	Spatial molecular mapping	~1–20 µm	Surface/section-based	Medium	Hours	Not required

**Table 4:** Translational and practical characteristics of imaging modalities.

Method	Advantages	Disadvantages	Limitations	Bedside Feasibility	Invasiveness	Approx. Cost	Clinical Readiness	Best Clinical/ Experimental Use Case
Light microscopy	High morphological detail; gold standard	Requires tissue processing	No live or dynamic imaging	No	Invasive	Low	Routine clinical use	Histopathology and morphology
IHC	High molecular specificity	Biopsy required; variability in staining	Semi-quantitative	No	Invasive	Low/moderate	Routine clinical use	Marker localization in tissue sections
SEM	Excellent surface topography	Complex preparation	Ex vivo only	No	Invasive	High	Research use	Surface ultrastructure analysis
TEM	Nanometer-scale detail	Labor-intensive	Very small field of view	No	Invasive	High	Research use	Ultrastructural analysis
WFFI	Fast, large-area imaging	No sectioning	Limited depth	Limited/selected settings	Non-invasive	Moderate	Established/adjunct use	Rapid screening and intraoperative imaging
CFM	Optical sectioning and higher contrast	Limited field of view	Shallow penetration	Limited	Non-invasive	Moderate/high	Established in selected applications	Margin assessment and local imaging
FLIM	Functional and metabolic information	Complex analysis	Costly and technically demanding	Limited	Non-invasive	High	Early translational/research	Metabolic phenotyping
MRI	Deep tissue imaging; noninvasive	High cost	Limited microscopic resolution	No	Non-invasive	High	Routine clinical use	Deep tissue and whole-organ imaging
Ultrasound	Portable and real-time	Operator dependent	Limited tissue contrast	Yes	Non-invasive	Low–moderate	Routine clinical use	Bedside diagnostics
MSI	Label-free profiling	Destructive; data-intensive	Slow and complex workflow	No	Destructive	High	Research/emerging translation	Molecular mapping of tissue sections

## Data Availability

No new data were created or analyzed in this study. Data sharing is not applicable to this article.
